# Mild electrical stimulation with heat shock guides differentiation of embryonic stem cells into Pdx1-expressing cells within the definitive endoderm

**DOI:** 10.1186/s12896-017-0331-z

**Published:** 2017-02-15

**Authors:** Tomoaki Koga, Nobuaki Shiraki, Shuichiro Yano, Mary Ann Suico, Saori Morino-Koga, Takashi Sato, Tsuyoshi Shuto, Shoen Kume, Hirofumi Kai

**Affiliations:** 1 Department of Molecular Medicine, Graduate School of Pharmaceutical Sciences, Kumamoto University, 5-1 Oe-honmachi, Chuo-ku, Kumamoto, 862-0973 Japan; 2 Present address: Department of Biochemistry, Juntendo University School of Medicine, 2-1-1 Hongo, Bunkyo-ku, Tokyo, 113-8421 Japan; 3 Department of Stem Cell Biology, Institute of Molecular Embryology and Genetics (IMEG), Kumamoto University, 2-2-1 Honjo, Chuo-ku, Kumamoto, 860-0811 Japan; 4 Present address: Department of Biological Information, School and Graduate School of Bioscience and Biotechnology, Tokyo Institute of Technology, 2-12-1 Ookayama, Meguro-ku, Tokyo, 152-8550 Japan

**Keywords:** Electrical stimulation, ES cells, Pancreatic β cells, Differentiation

## Abstract

**Background:**

Because of the increasing number of diabetic patients, it is important to generate pancreatic and duodenal homeobox gene 1 (Pdx1)-expressing cells, which are capable of differentiating into pancreatic endocrine β cells. Mild electrical stimulation was reported to modulate the differentiation of ES cells into ectoderm-derived neuronal cells or mesoderm-derived cardiac cells.

**Results:**

In this study, we report that mild electrical stimulation with heat shock (MET) potentiates the differentiation of ES cells into definitive endoderm-derived Pdx1-expressing cells. MET has no effect when applied to early definitive endoderm on differentiation day 5. A 1.87-fold increase in the proportion of Pdx1-expressing cells was observed when stimulation was applied to the late definitive endoderm one day prior to the immergence of *Pdx1*/GFP-expressing cells on differentiation day 7. *Pdx1* mRNA was also up-regulated by MET. The potentiating effect of MET synergized with activin and basic fibroblast growth factor into Pdx1-expressing cells. Moreover, MET stimulation on late definitive endoderm up-regulated heat shock protein 72 and activated various kinases including Akt, extracellular signal-regulated kinase, p38, and c-jun NH_2_-terminal kinase in ES cells.

**Conclusions:**

Our findings indicate that MET induces the differentiation of Pdx1-expressing cells within the definitive endoderm in a time-dependent manner, and suggest useful application for regenerative medicine.

**Electronic supplementary material:**

The online version of this article (doi:10.1186/s12896-017-0331-z) contains supplementary material, which is available to authorized users.

## Background

Loss or insufficiency of insulin-producing pancreatic β cells is a critical issue for type 1 and type 2 diabetic patients [1, 2]. Transplantation of islets has been expected as a complete cure for diabetes mellitus [3]. However, it is difficult to supply sufficient β cells to cure patients because of insufficiency of donors. In addition, due to the growing number of diabetic patients, the development of novel strategies to produce β cells is urgently needed. Because of their pluripotency, embryonic stem (ES) cells are expected to be a viable source of β cells. Many strategies utilizing growth factors and signaling pathways have been reported to initiate differentiation and modulate the course of cellular differentiation [4].

Mechano-stress such as shear stress, stretch stress, and electrical stress has been reported to modulate various intracellular signaling pathways [5, 6]. Of those, electrical stimulation has been recently known to modulate fate determination of differentiating mouse ES cells into neuronal cells, which are of ectoderm origin through Ca^2+^ influx, and human ES cells into cardiac cells, which are of mesoderm origin, through reactive oxygen species (ROS) production [7, 8]. While the effects of electrical stimulation on the differentiation of ES cells into ectoderm and mesoderm are known, its effect on the differentiation of ES cells into pancreatic lineage, which is of definitive endoderm origin, remains unclear. We previously demonstrated that mild electrical stimulation with heat shock (MET) stimulation synergistically ameliorates insulin resistance *via* enhanced insulin signaling in L6 skeletal muscle cells and hepatic HepG2 cells in vitro, and high fat diet or *db*/*db* diabetic mice in vivo [9–11]. We have shown that MET was more effective than mild electrical stimulation or heat shock alone. Therefore, we investigated the effect of combination treatment of mild electrical stimulation and heat shock on the ES cell differentiation into pancreatic lineage.

## Results

### MET stimulation on day 5 does not affect the differentiation of definitive endoderm or Pdx1-expressing cells

**Fig. 1:**
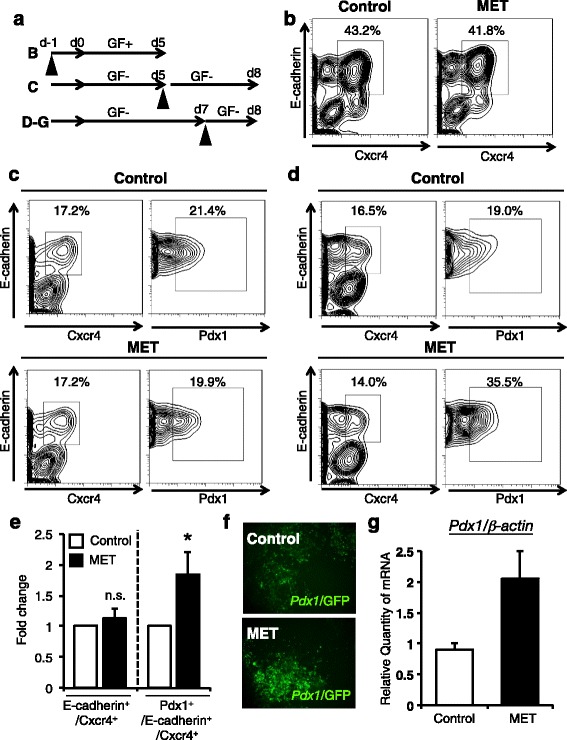
MET stimulation on day 7 enhances the generation of Pdx1-expressing cells derived from mouse ES cells but not stimulation on day -1 or day 5*.* **a** Experimental flow of MET on ES cell differentiation into definitive endoderm (d5) and Pdx1-expressing cells within the definitive endoderm (d8). GF; growth factors (activin and bFGF). Closed arrowheads indicate MET stimulation. **b** SK7 ES cells were stimulated by MET on day -1 followed by FACS analysis on day 5. Numbers indicate the proportion of E-cadherin+/Cxcr4+ definitive endoderm cells within total ES cell culture. **c** ES cells were treated with MET on day 5 followed by FACS analysis on day 8. Numbers indicate the proportion of E-cadherin+/Cxcr4+ definitive endoderm cells within total ES cell culture (*left panels*). Numbers indicate the proportion of E-cadherin+/Pdx1+ pancreatic progenitor cells within the definitive endoderm cells (*right panels*). Upper panels are sham-treated controls and bottom panels are MET-treated samples. Differentiation was done without any additional growth factors. **d** ES cells were stimulated by MET on day 7 followed by FACS analysis on day 8. Numbers indicate the proportion of E-cadherin+/Cxcr4+ definitive endoderm cells within total ES cell culture (*left panels*). Numbers indicate the proportion of E-cadherin+/Pdx1+ pancreatic progenitor cells within definitive endoderm cells (*right panels*). **e** Percentage of differentiated cells/ total cells on Day 8 treated with MET on Day 7 was normalized with sham-treated control and indicated as fold change as assessed by flow cytometry (*n* = 3, Error bars indicates ± SEM. *p* = 0.041). **f** Representative fluorescent images of ES cells on Day 8. **g** MET stimulation likely induces the expression of *Pdx1* mRNA expression in SK7 ES cells (*p* = 0.128). β-actin was used as internal control. Values are the mean ± S.E. from triplicate plates for E and duplicate plates for **g**. Statistical significance was determined by Student’s *t*-test. *; *p* < 0.05, n.s.; not significant

To investigate whether MET stimulation affects ES cell differentiation into pancreatic progenitor cells, SK7 ES cells were plated on M15 feeder cells. The cell set-up and MET treatment is shown (Additional file 1: Figure S1A, B). We first treated ES cells with MET for 10 min on the day before starting differentiation (day -1). Cells were subjected to flow cytometry on day 5 to determine the proportion of E-cadherin+/Cxcr4+ definitive endoderm (Fig. 1a, *upper*). As shown in Fig. 1b, MET stimulation had no effect on the differentiation into definitive endoderm. We next stimulated cells with MET on day 5 after starting differentiation. Then cells were analyzed on day 8 to assess E-cadherin+/Cxcr4+ definitive endoderm and *Pdx1*/GFP-expressing cells within the definitive endoderm, which are pancreatic β cell progenitor cells expressing the earliest marker pancreatic and duodenal homeobox gene 1 (Pdx1) [12] (Fig. 1a, *middle*). MET stimulation had no effect on the differentiation into the definitive endoderm and *Pdx1*/GFP-expressing cells within the definitive endoderm (Fig. 1c). Taken together, MET stimulation on day -1 or day 5 has no effect on the differentiation into the definitive endoderm or Pdx1-expressing cells.

### MET stimulation on day 7 potentiated the differentiation of ES cells into Pdx1-expressing cells within the definitive endoderm

We next determined whether MET stimulation affects the differentiation of ES cells into E-cadherin+/Cxcr4+ definitive endoderm or *Pdx1*/GFP-expressing cells within the definitive endoderm at later stage of differentiation. ES cells were stimulated with MET on day 7 prior to the appearance of *Pdx1*/GFP-expressing cells within the definitive endoderm, and analyzed on day 8 by flow cytometry (Fig. 1a, *lower*). MET treatment on day 7 had no effect on definitive endoderm population similar to day 5 stimulation. Interestingly, however, the proportion of *Pdx1*/GFP-expressing cells within the definitive endoderm population was increased 1.87-fold in MET-treated groups compared with control (Fig. 1d). This enhancement of the *Pdx1*/GFP-expressing cells within the definitive endoderm population was statistically significant (Fig. 1e). Increase of *Pdx1*/GFP-expressing cells was also confirmed by fluorescent microscopy (Fig. 1f). Endogenous expression of *Pdx1* mRNA was assessed by Q-PCR analysis. Although it was not statistically significant, *Pdx1* mRNA expression tended to be induced by MET stimulation (Fig. 1g). Collectively, MET stimulation on day 7 potentiated the differentiation of ES cells into *Pdx1*/GFP-expressing cells within the definitive endoderm.

### MET stimulation on day 7 potentiated the differentiation of ES cells into Pdx1-expressing cells within the definitive endoderm induced by activin and bFGF treatment

**Fig. 2:**
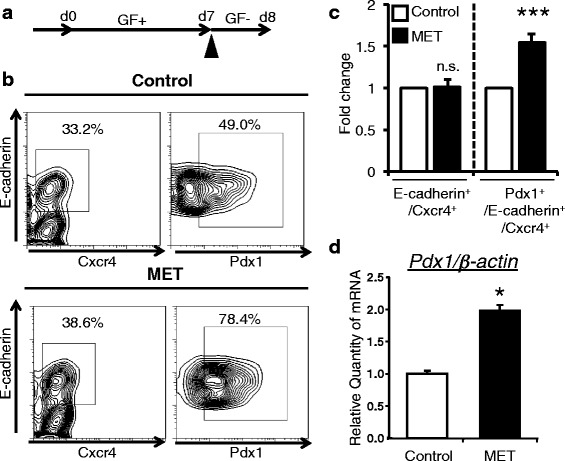
MET stimulation enhances the generation of Pdx1-expressing cells derived from mouse ES cells induced by activin and bFGF. **a** Experimental flow of MET on ES cell differentiation into Pdx1-expressing cells within the definitive endoderm (d8). GF; growth factors (activin and bFGF). *Closed arrowhead* indicates MET stimulation. **b** ES cells were stimulated by MET on day 7 followed by FACS analysis on day 8. Numbers indicate the proportion of E-cadherin+/Cxcr4+ definitive endoderm cells within total ES cell culture (*left panels*). Numbers indicate the proportion of E-cadherin+/Pdx1+ pancreatic progenitor cells within definitive endoderm cells (*right panels*). **c** Percentage of differentiated cells/ total cells on Day 8 co-treated with MET, activin and bFGF was normalized with sham-treated control and indicated as fold change as assessed by flow cytometry (*n* = 6, Error bars indicates ± SEM. *p* = 0.00023). **d** MET stimulation enhances the expression of *Pdx1* mRNA expression in SK7 ES cells. β-actin was used as internal control (*p* = 0.049). Values are the mean ± S.E. from 6 plates for **c** and 2 plates for **d**. Statistical significance was determined by Student’s *t*-test. *; *p* < 0.05, ***; *p* < 0.001, n.s.; not significant

We previously reported that activin and basic fibroblast growth factor (bFGF) guide the differentiation of ES cells into *Pdx1*/GFP-expressing cells in definitive endoderm [4]. Here, we investigated whether MET treatment potentiates the growth factors-induced differentiation of ES cells into *Pdx1*/GFP-expressing cells. ES cell differentiation was performed in the presence of activin and bFGF. MET stimulation was done on day 7 before the expression of *Pdx1*/GFP was detected. Cells were analyzed on day 8 by flow cytometry (Fig. 2a). As shown in Fig. 2b, MET enhanced the differentiation of ES cells into *Pdx1*/GFP-expressing regional-specific definitive endoderm populations, but had no effect on definitive endoderm differentiation. This enhancement of the *Pdx1*/GFP-expressing cells within the definitive endoderm population was statistically significant (Fig. 2c). In addition, endogenous expression of Pdx1 mRNA was also increased in MET-stimulated groups assessed by Q-PCR analysis (Fig. 2d). Moreover, MET treatment enhances the mRNA expression of FoxA2, an important transcription factor for the differentiation of pancreas and liver (Additional file 2: Figure S2). Taken together, MET stimulation potentiates the activin and bFGF-induced differentiation of ES cells into Pdx1-expressing cells within the definitive endoderm.

### MET stimulation on day 7 up-regulated Hsp72 expression and activated various kinases in mouse ES cells

**Fig. 3:**
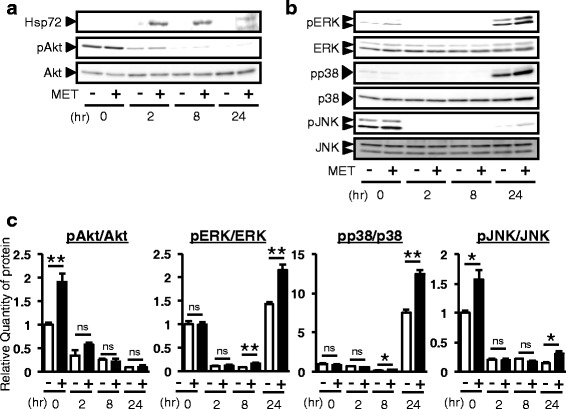
MET stimulation on day 7 up-regulates the expression of Hsp72 and activates various signaling pathways in mouse ES cells. **a**, **b**, **c** SK7 ES cells were differentiated into Pdx1-expressing cells by activin and bFGF from day 0 to day 7. On day 7 after starting the differentiation, MET stimulation was performed, and activin and bFGF were removed by changing the medium. Protein lysates were collected at the indicated time. pAkt; phosphorylated Akt, pERK; phosphorylated ERK, pp38; phosphorylated p38, pJNK; phosphorylated JNK. Densitometric analysis was done with ImageGauge software. Density of phosphorylated proteins was normalized by that of total proteins. Statistical significance was determined by Student’s *t*-test. *; *p* < 0.05, **; *p* < 0.01, n.s.; not significant. Specific p values (pAkt/Akt: *p* = 0.0089, pERK/ERK: *p* = 0.002 for 8 h, *p* = 0.006 for 24 h, pp38/p38: *p* = 0.019 for 8 h, *p* = 0.002 for 24 h, pJNK/JNK: *p* = 0.025 for 0 h, *p* = 0.036 for 24 h)

We investigated how MET stimulation on day 7 potentiates the differentiation of ES cells into *Pdx1*/GFP-expressing cells within the definitive endoderm induced by activin and bFGF treatment. Because we previously reported that MET stimulation up-regulates Hsp72 expression and activates Akt signaling in hepatocyte and skeletal muscle cell line, we assessed whether MET induces Hsp72 and activates Akt signaling in ES cells. SK7 ES cells were stimulated by MET on day 7 after starting the differentiation in the presence of activin and bFGF. MET induced the expression of Hsp72 in ES cells from 2 to 24 h after stimulation (Fig. 3a). Akt phosphorylation was enhanced immediately after MET treatment (Fig. 3a, b). Because mitogen-activated protein kinases (MAPKs) are known to regulate the differentiation of ES cells into Pdx1-expressing cells [4], we next examined whether MET activates MAPKs signaling pathways in ES cells. ES cell protein lysates were subjected to Western blot analysis. MET activated JNK immediately after stimulation, whereas ERK and p38 MAPKs were activated at 24 h after MET stimulation (Fig. 3b, c). Collectively, MET stimulation on day 7 induces the expression of Hsp72 and activates various kinases including Akt, ERK, p38 and JNK.

## Discussion

Recent reports showed that electrical stimulation enhances the differentiation of ES cells into neural cells in ectoderm or cardiac cells in mesoderm [7, 8]. However, the impact of electrical stimulation on Pdx1-expressing pancreatic progenitor cells in definitive endoderm remains unknown. We firstly identified MET stimulation as a novel stimulus to induce ES cell differentiation into Pdx1-expressing cells within the definitive endoderm (Fig. 1d-g). To our knowledge, this is the first report showing that applied electrical signal affects the differentiation of ES cells into Pdx1-expressing cells. In addition, co-stimulation with MET and growth factors resulted in differentiating ES cells into Pdx1 cells more efficiently, suggesting an important contribution of MET to the development of Pdx1-expressing cells (Fig. 2). Furthermore, as FoxA2 was up-regulated by MET treatment, it is possible that MET also potentiates liver differentiation. Future investigations on the effect of MET on FoxA2 will clarify a possible application of MET in this area. Because MET stimulation is easy to use and economical, our findings also contain important implications with respect to macro-scale industrialization of regenerative medicine.

Another interesting issue is that MET stimulation on late definitive endoderm on day 7 enhances the differentiation of ES cells into Pdx1-expressing cells within the definitive endoderm population, whereas MET stimulation on day -1 (undifferentiated cells) or day 5 early definitive endoderm cells had no effect (Fig. 1b, c). Recent report showed that gene expression profiles of differentiated ES cells are totally different between day 5 and day 7 definitive endoderm populations [13]. Although detailed molecular mechanisms still have to be investigated, it is possible that at day 7 late definitive endoderm, cells are able to sense MET stimulation as differentiation signals but not the day -1 undifferentiated cells or day 5 early definitive endoderm cells.

We previously reported that MET stimulation activates phosphatidylinositol-3-kinase (PI3K)-Akt signaling and up-regulates the expression of Hsp72 [9–11, 14, 15]. PI3K-Akt signaling is crucial in the self-renewal of ES cells, and the Pdx1 up-regulation and differentiation from pancreatic ductal cells [16, 17]. Moreover, Hsp72 is known as an anti-apoptotic molecular chaperone and to control the activation of Smad3, an important downstream molecule of activin signaling [18]. The up-regulation of Hsp72 and the activation of Akt were observed in ES cells stimulated by MET (Fig. 3a, c). In addition, ERK, p38, and JNK MAPKs were activated by MET (Fig. 3b, c). Because Akt, ERK, JNK, and p38 are important downstream molecules of bFGF signaling [19], and p38 is a critical downstream kinase of activin signaling [20], we propose that MET activates these signaling pathways and subsequently induces ES cell differentiation into Pdx1-expressing cells. Future studies will focus on the identification of molecules involved in sensing MET stimulation as stimuli and transducing it to intracellular signaling pathways to potentiate the differentiation of ES cells into Pdx1-expressing cells within the definitive endoderm.

## Conclusions

Collectively, we reported in this study a novel procedure for inducing pancreatic progenitor cells, which express the earliest marker Pdx1. MET stimulation enhances the differentiation of ES cells into Pdx1-expressing pancreatic progenitor cells within the definitive endoderm. MET treatment synergized with activin and bFGF growth factors in differentiating ES cells into pancreatic progenitor cells. Our findings indicated that MET stimulation has beneficial effects on the differentiation of Pdx1-expressing cells within the definitive endoderm and suggest important application for regenerative medicine.

## Methods

### Cell culture and differentiation

The ES cell line SK7 containing a *Pdx1* promoter-driven GFP reporter transgene was established and maintained as described previously [4, 21]. The mesonephric cell line M15 was used as feeder cell for pancreatic differentiation [4]. SK7 cells were maintained on mouse embryonic fibroblast (MEF) feeders in Glasgow minimum essential medium (Invitrogen, Carlsbad, CA) lemented with 1,000 units/mL leukemia inhibitory factor (LIF; Chemicon, Temecula, CA), 15% Knockout Serum Replacement (KSR; Gibco, Grand Island, NY), 1% fetal bovine serum (FBS; HyClone, Logan, UT), 100 μM nonessential amino acids (NEAA; Invitrogen), 2 mM L-glutamine (L-Gln; Invitrogen), 1 mM sodium pyruvate (Invitrogen), 50 units/ml penicillin and 50 μg/ml streptomycin (PS; Invitrogen), and 100 μM β-mercaptoethanol (β-ME; Sigma-Aldrich, St. Louis). For differentiation studies, ES cells were plated at 50,000 cells per dish in 60 mm dishes (Falcon) that had been previously coated with M15 cells. The cells were cultured in differentiation medium (DMEM supplemented with 10% FBS, 4500 mg/L glucose, NEAA, L-Gln, PS and β-ME) for 8 days. Medium was changed every other day. For the activin and bFGF-induced differentiation study, activin (10 ng/ml) and bFGF (5 ng/ml) were removed after MET stimulation to determine the effect of MET stimulation on differentiation.

### Real-time quantitative PCR (Q-PCR) analysis

Total RNA was collected from differentiated ES cells using TRIzol reagent (Invitrogen) according to manufacturer’s instructions. Real time quantitative RT-PCR analysis for Pdx1 and β-actin were carried out using PrimeScript RT reagent kit (TaKaRa) and SYBR Premix Ex Taq™ II (TaKaRa). PCR amplifications were performed as described previously [4]. The threshold cycle values for Pdx1 amplification was normalized by subtracting the threshold cycle value calculated for β-actin (internal control). The normalized gene expression values were calculated (e^-ΔCt) as the relative quantity of gene-specific expression (e = 1.956 for mPdx1). Pdx1 mRNA expression was indicated as a fold induction against sham-treated control. The following primers were used for *Pdx1*: Forward, 5′-CCAAAACCGTCGCATGAAGTG-3′ and Reverse, 5′-CTCTCGTGCCCTCAAGAATTTTC-3′, and for *β-actin*: Forward, 5′-GTGATGGTGGGAATGGGTCA -3′ and Reverse, 5′-TTTGATGTCACGCACGATTTCC-3′.

### Mild electrical stimulation with heat shock (MET) treatment

ES cells were plated on M15 cells in 60-mm culture dishes and were treated with MET as described previously [10, 11]. Briefly, flat rubber electrodes connected to a BioMetronome™ (Tsuchiya Gum Co., Ltd., Kumamoto, Japan) were put into the culture media and MET stimulation was delivered using 1 V/cm (55 pps) of direct current with individual pulse duration of 0.1 ms (Additional file 1: Figure S1A). The culture plate with the electrodes was carefully sealed and immersed in a water bath at 42 °C. Stimulation was performed for 10 min.

### Flow cytometry

The following antibodies were used: biotin-conjugated anti-E-cadherin mAb ECCD2 [22], phycoerythrin (PE)-conjugated anti-Cxcr4 mAb 2B11, and streptavidin-APC (BD Biosciences, San Diego). The stained cells were analyzed with a FACSCanto (BD Biosciences). Data were analyzed by FlowJo program (Tree Star, Ashland, OR).

### Immunoblotting

Immunoblotting were performed as described previously [9, 10]. Briefly, protein lysates from ES cells were isolated with lysis buffer (25 mM HEPES, 10 mM Na_4_P_2_O_7_・10H_2_O, 100 mM NaF, 5 mM EDTA, 2 mM Na_3_VO_4_, 1% Triton X-100). Protein lysates were subjected to SDS-PAGE and Western blotting. The blots were probed with the indicated antibodies and their respective HRP-conjugated secondary antibodies obtained from JacksonImmuno Research Laboratories (West Grove, PA). Antibody against Hsp72 was purchased from Stressgen Biotechnologies (Victoria, BC, Canada). Antibodies against phospho-Akt, Akt, phospho-extracellular signal-regulated kinase (ERK), ERK, phospho-p38, p38, phospho-c-jun NH_2_-terminal kinase (JNK), and JNK were purchased from Cell Signaling Technology (Beverly, MA). Densitometric analysis was performed by ImageGauge software.

### Statistical analysis

Data are presented as the mean ± S.E. Significance of the differences between groups were assessed with Student’s unpaired two-tailed *t*-test. A *P* value of <0.05 was considered statistically significant.

## Additional files

Additional file 1: Figure S1. Diagrams of the apparatus for MET stimulation and differentiation flow of ES cells on M15 feeder cells. (A) Diagram of MET treatment in vitro. MES: mild electrical stimulation. ms; millisecond, pps; pulses per second. (B) Differentiation flow of ES cells into Pdx1-expressing cells within the definitive endoderm on M15 feeder cells. Ect: ectoderm, ME: mesoendoderm, Mes: mesoderm, End: endoderm, Pdx1: Pdx1-expressing cells. (PDF 44 kb)
Additional file 2: Figure S2. Effect of MET treatment on the expression of Foxa2 mRNA. MET stimulation enhances the expression of *mFoxa2* mRNA expression in SK7 ES cells. b-actin was used as internal control (*p* = 0.031). Values are the mean ± S.E. Statistical significance was determined by Student’s *t*-test. *; *p* < 0.05. (PDF 36 kb)

## Abbreviations

ES cells: Embryonic stem cells
MET: Mild electrical stimulation with heat shock
Pdx1: Pancreatic and duodenal homeobox gene 1
