# Supplementary figures and images for: Mild electrical stimulation with heat shock guides differentiation of embryonic stem cells into Pdx1-expressing cells within the definitive endoderm
Source: BMC Biotechnol. 2017 Feb 15;17:14. doi: 10.1186/s12896-017-0331-z (PMC5312430; doi:10.1186/s12896-017-0331-z)

A.

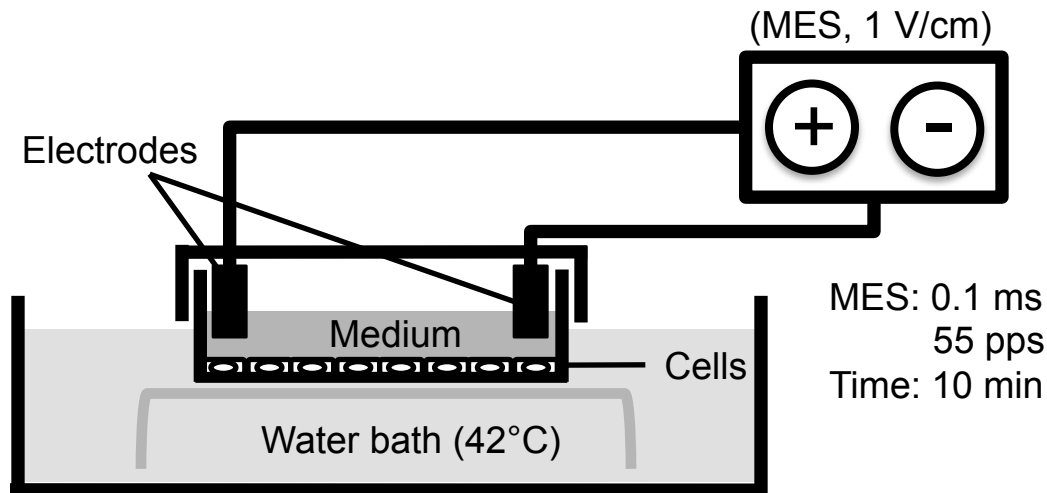

B.

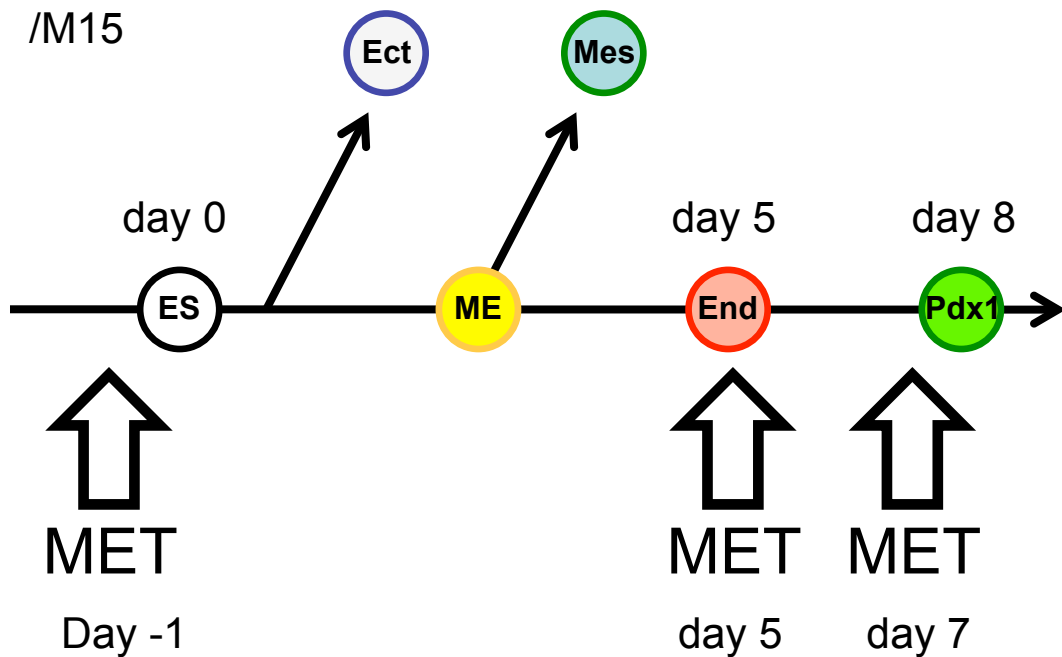

Supplement: Additional file 1: Figure S1. — Diagrams of the apparatus for MET stimulation and differentiation flow of ES cells on M15 feeder cells. (A) Diagram of MET treatment in vitro. MES: mild electrical stimulation. ms; millisecond, pps; pulses per second. (B) Differentiation flow of ES cells into Pdx1-expressing cells within the definitive endoderm on M15 feeder cells. Ect: ectoderm, ME: mesoendoderm, Mes: mesoderm, End: endoderm, Pdx1: Pdx1-expressing cells. (PDF 44 kb) [file 12896_2017_331_MOESM1_ESM.pdf]

*Foxa2/β-actin*

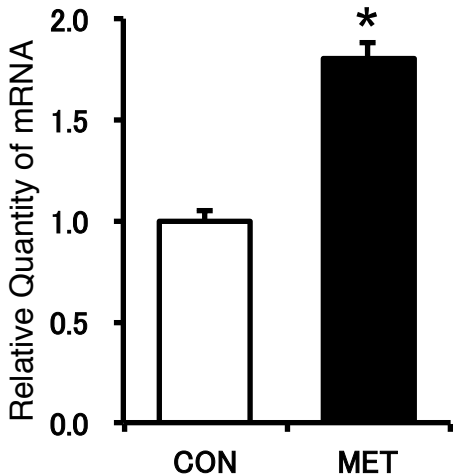

Supplement: Additional file 2: Figure S2. — Effect of MET treatment on the expression of Foxa2 mRNA. MET stimulation enhances the expression of mFoxa2 mRNA expression in SK7 ES cells. b-actin was used as internal control (p = 0.031). Values are the mean ± S.E. Statistical significance was determined by Student’s t-test. *; p < 0.05. (PDF 36 kb) [file 12896_2017_331_MOESM2_ESM.pdf]
